# The improvement of *Hovenia acerba*-sorghum co-fermentation in terms of microbial diversity, functional ingredients, and volatile flavor components during Baijiu fermentation

**DOI:** 10.3389/fmicb.2023.1299917

**Published:** 2024-01-05

**Authors:** Jing Zhang, Minhui Zhao, Jing Chen, Yuanting Zhu, Chen Xiao, Qi Li, Xiaoqi Weng, Yunxuan Duan, Yong Zuo

**Affiliations:** ^1^College of Food and Biological Engineering, Chengdu University, Chengdu, China; ^2^Solid-state Fermentation Resource Utilization Key Laboratory of Sichuan Province, Faculty of Quality Management and Inspection and Quarantine, Yibin University, Yibin, China; ^3^College of Life Science, Sichuan Normal University, Chengdu, China

**Keywords:** microbial community, organic acids, polyphenols, volatile flavor components, correlation analysis

## Abstract

The quality of Baijiu was largely affected by raw materials, which determine the flavor and taste. In the present study, organic acids, polyphenols, volatile flavor components and microbial community in *Hovenia acerba*-sorghum co-fermented Baijiu (JP1) and pure sorghum-fermented Baijiu (JP2) were comprehensively analyzed. Organic acids, polyphenols and volatile flavor components in JP1 were more abundant than JP2. The abundance and diversity of bacteria and fungi in JP1 was higher than that in JP2 in the early stage of fermentation, but presented opposite trend in the middle and late stages. *Leuconostoc*, *Lentilactobacillus* and *Issatchenkia* were dominant genera in JP1. Whereas, Cronobacter, Pediococcus and Saccharomyces occupied the main position in JP2. *Lentilactobacillus* and *Issatchenkia* were positively related to most of organic acids and polyphenols. *Pseudomonas, Rhodococcus, Cronobacter, Pediococcus, Brucella, Lentilactobacillus, Lactobacillus, Saccharomycopsis, Wickerhamomyces, Aspergillus, Thermomyces* and *unclassified_f—Dipodascaccae* were associated with the main volatile flavor components. The main metabolic pathways in two JPs exhibited the variation trend of first decreasing and then increasing, and the metabolism activity in JP1 were higher than that in JP2. The results demonstrated the introduction of *Hovenia acerba* improved the functional ingredients and volatile flavor components, which is helpful for the quality promotion of Baijiu. This study identified the key microorganisms and discussed their effect on organic acids, polyphenols and volatile flavor components during the fermentation of Baijiu with different raw materials, providing a scientific basis for the development and production of high-quality Baijiu.

## 1 Introduction

Baijiu, fermented from sorghum, corn, and other grains and with a brewing history of more than 2,000 years, fulfills a typical element of Chinese traditional culture (Li et al., 2017; Sha et al., 2017). Due to the differences in raw materials, the nutritional ingredients and volatile flavor components vary, further influencing the taste and quality of Baijiu. With the improvement in living standards, consumers pay more attention to the quality of products, and health has become the focus. However, Baijiu fermented from sole raw material was weak in flavor and nutrition. Additionally, present research on Baijiu mainly focuses on flavor, neglecting the functions of nutritional ingredients and their importance to health (Liu et al., 2012; Xiao et al., 2014; Zhao et al., 2018). Therefore, the development of the Baijiu industry should adhere to the dual orientation of flavor and health.

*Hovenia acerba*, a kind of tall tree of the Rhamnaceae family, is a source of ingredients including polysaccharides, amino acids, flavonoids, and fatty acids (Wang et al., 2017), providing functions such as liver protection, hypoglycemia, and anti-oxidation (Zhou et al., 2013). As a medicinal and edible plant, *Hovenia acerba* is widely applied in medicine, healthcare, and the food industry (Ma and Zhou, 2022). In recent years, as a kind of raw material, *Hovenia acerba* has been used in the production of Baijiu alongside sorghum (Minhui et al., 2023). Although research on raw material treatment and brewing technology optimization has been conducted to improve the quality of *Hovenia acerba*-sorghum co-fermented Baijiu, systematic discussions of nutritional ingredients and volatile flavor components and their correlation with the microbial community have not been reported.

With the rapid development of modern molecular biology and detection technology, high-throughput sequencing, gas chromatography–mass spectrometry (GC–MS), and high-performance liquid chromatography (HPLC) have been widely applied in the study of microbial diversity and flavor substances during the fermentation of Baijiu, enhancing the understanding of microbial community composition and characteristic flavor (Cai et al., 2021; Qian et al., 2021). The correlation between the microbial community and the flavor substances of strong aromatic Baijiu was studied by Zhang et al. Water and alcohol content had a great impact on the production of flavor compounds such as ethyl lactate, caproic acid, and ethyl caproate. Six bacterial genera and seven fungal genera exhibited a significant influence on caproic acid (Zhang et al., 2021). Based on redundancy analysis and the Mantel test, Lin et al. studied the correlation between microorganisms and metabolic substances during the fermentation of Xiaoqu Baijiu. The content of water, amino acid nitrogen, acids, and reducing sugar were significantly related to bacteria and fungi. *Pichia*, *Rhizopus*, *Saccharomyces*, and *Wickerhamomyces* were significantly related to the main alcohols, esters, and aldehydes, playing important roles in the formation of the flavor of Xiaoqu Baijiu (Li et al., 2022). The studies mentioned above provide reliable means for us to understand the succession of microbial communities and changes in flavor components in the process of Baijiu brewing. It is helpful for the correlation analysis of the microbial community, flavor substances, and bioactive components during the fermentation of *Hovenia acerba*-sorghum co-fermented Baijiu and conducive to in-depth research of its brewing mechanism.

In this study, the dynamics of physicochemical properties, organic acids, polyphenols, volatile flavor components, and microbial community during the fermentation of *Hovenia acerba*-sorghum co-fermented Baijiu and pure sorghum-fermented Baijiu were overall compared by combining physicochemical analysis, GC–MS, HPLC, and high-throughput sequencing. The interaction networks of the microbial community and their relationship with organic acids, polyphenols, and volatile flavor components were also revealed.

## 2 Materials and methods

### 2.1 Sample preparation and collection

Sample preparation: the brewing technology of *Hovenia acerba*-sorghum co-fermented Baijiu was as follows:

Key points: (1) *Hovenia acerba* was cut into 1 cm segments using scissors and steamed for 40 min in a steamer. (2) Sorghum was crushed, then soaked in 70°C water for 1 h, and steamed for another hour. After that, sorghum was mixed with 40% sterile water (w/w), blended well, and cooled to room temperature. (3) 20% *Hovenia acerba* and 80% sorghum (w/w) were mixed with Daqu and transferred into a fermentation barrel for airtight fermentation at 28°C. Pure sorghum Baijiu was prepared using a similar method mentioned above by using sorghum as the sole raw material.

Sample collection: The fermented grain of *Hovenia acerba*-sorghum co-fermented Baijiu (marked as JP1) and the pure sorghum-fermented Baijiu (marked as JP2) were collected on the 0, 7, 14, 21, 28, and 35th days of fermentation.

### 2.2 Determination of volatile flavor components

The determination of volatile flavor components was carried out according to the report with minor modifications (Yang et al., 2023).

Headspace solid phase microextraction (HS-SPME): A total of 5 mL of sample was added into the headspace bottle with 2.5 g NaCl and 20 μL internal standard (2-octanol, 450 μg/mL). The bottle was heated in a 50°C thermostatic water bath for 5 min. The activated and balanced extraction head (aging at 250°C for 15 min) was inserted into the headspace bottle for 45 min.

GC condition: The chromatographic separation was performed on the DB-WAX Capillary column (60 m × 250 μm × 0.25 μm). Temperature procedure was as follows: (1) the initial temperature was kept at 50°C for 2 min; (2) it was increased to 60°C at a rate of 2°C/min and held for 1 min; (3) it was increased to 105°C at a rate of 3°C/min and held for 3 min; (4) it was increased to 180°C at a rate of 4°C/min and held for 3 min; and (5) it was increased to 230°C at a rate of 6°C/min and held for 1 min. The injection port temperature was 230°C. The carrier gas was highly pure He (99.999%) with a flow rate of 1 mL/min and no split flow.

MS condition: The MS was conducted in an electron ionization (EI) at 70 eV. The ion source temperature was 230°C. The interface temperature was 230°C.

### 2.3 Detection of organic acids

The detection of organic acids was performed according to Zhang’s description (Zhang et al., 2023).

A total of 5 g of fermented grain was added to 20 mL of ultrapure water and then shaken and mixed well. Then, the sample was sonicated for 30 min and centrifuged at 7104 × *g* at 4°C for 5 min. The supernatant was obtained for further use after passing through the 0.22 μm nylon filter membrane.

HPLC conditions: The chromatographic separation was performed on an Agilent ZORBAX SB-Sq column (4.6 mm × 250 mm, 5 μm). The column temperature was 30°C. The mobile phase consisted of 95% KH_2_PO_4_ (0.05 M, pH = 2.54) and 5% methanol with a flow rate of 0.4 mL/min. The injection volume was 20 μL. The detection wavelength was 210 nm.

Qualitative and quantitative analysis: The standard solution of oxalic acid, tartaric acid, pyruvic acid, malic acid, lactic acid, citric acid, acetic acid, succinic acid, and fumaric acid was prepared with KH_2_PO_4_ solution. The qualitative analysis of organic acids was conducted by referring to the retention time of standards. The quantitative analysis of organic acids was carried out by calculating the peak area.

### 2.4 Detection of polyphenols

Polyphenols were detected by referring to Valero-Cases’s report (Valero-Cases et al., 2017).

The polyphenols were analyzed using high-performance liquid chromatography. 50 mg of fermented grain was added to a 10 mL 70% methanol solution and extracted using ultrasonication for 30 min, followed by centrifugation at 7104 × *g* for 15 min at 4°C. The supernatant was obtained by passing it through the 0.22 μm filter membrane.

HPLC conditions: The chromatographic separation was performed on an Agilent ZORBAX SB-Sq column (4.6 mm × 250 mm, 5 μm). The column temperature was 30°*C. mobile* phase A: 1% formic acid. Mobile phase B: 100% acetonitrile. The flow rate was 1 mL/min. The injection volume was 10 μL. The detection wavelength was 280 nm. The gradient elution procedure is shown in Supplementary Table S1.

In total, 10 mg of quercetin, dihydromyricetin, epicatechin, catechin, chlorogenic, vanillic acid, isovanillic acid, syringic acid, p-coumaric acid, rutin, caffeic acid, and ferulic acid were weighed separately and dissolved in 5 mL methanol (70%). The solution was then transferred into a 10-mL volumetric flask.

The standard solution was diluted step by step (50, 75, 100, 150, 250, and 500 μg/mL) and analyzed according to the chromatographic conditions. The standard curve and the regression equation for polyphenols were established by fitting the peak area and concentration of the standard substance. The qualitative analysis of polyphenols was conducted according to the retention time, and the quantitative analysis was carried out according to the peak area.

### 2.5 Microbial community structure

The community structure of bacteria and fungi in JP1 and JP2 was identified by high-throughput sequencing by referring to Jiang’s report (Jiang et al., 2023). For bacteria, the amplification region was the V3-V4 region with primers 338F (5′-ACTCCTACGGGAGGCAGCAG-3′) and 806R (5′-GGACTACHVGGGTWTCTAA-3′). The amplification region was ITS1 for fungi, and the primers were ITS1F (5′-CTTGGTCATTTAGAGGAAGTAA-3′) and ITS2R (5′-GCTGCGTTCTTCATCGATGC-3′). The high-throughput sequencing was conducted by Shanghai Meiji Biomedical Technology Co., Ltd., mainly including total DNA extraction, sample quality inspection, PCR amplification, product purification, library construction, and Illumina sequencing.

### 2.6 Data analysis

All experiments were repeated three times, and the results were expressed as mean ± standard deviations. Analysis of variance (ANOVA) was performed using SPSS 20.0 to evaluate the significance level. Pearson’s correlation analysis between microbial composition and biochemical components was performed by Origin2021.

## 3 Results and discussion

### 3.1 Physicochemical properties

**Figure 1:**
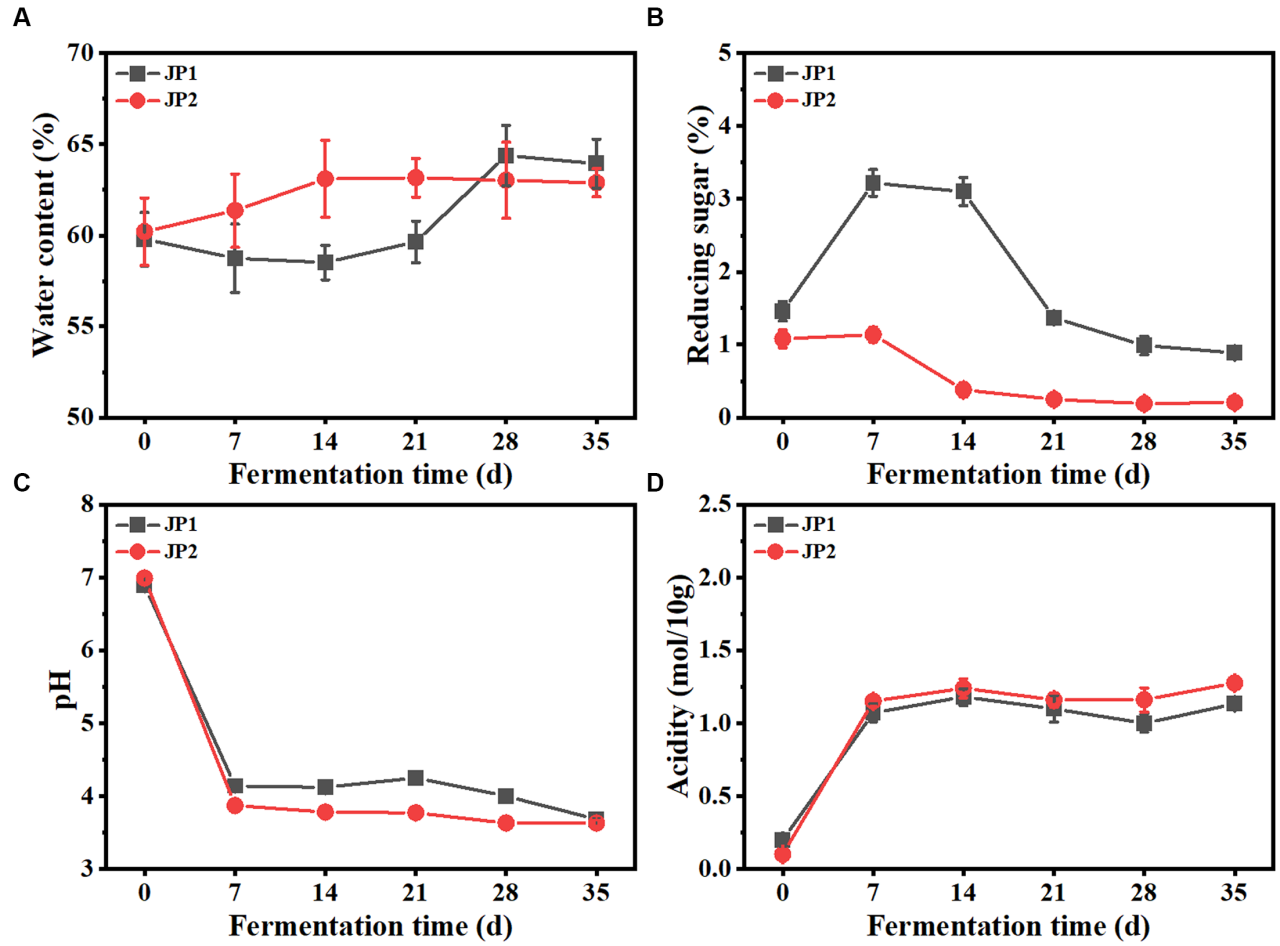
Dynamics of physicochemical properties during the fermentation of JPs. **(A)** Water content. **(B)** Reducing sugar. **(C)** pH. **(D)** Acidity.

**Figure 2:**
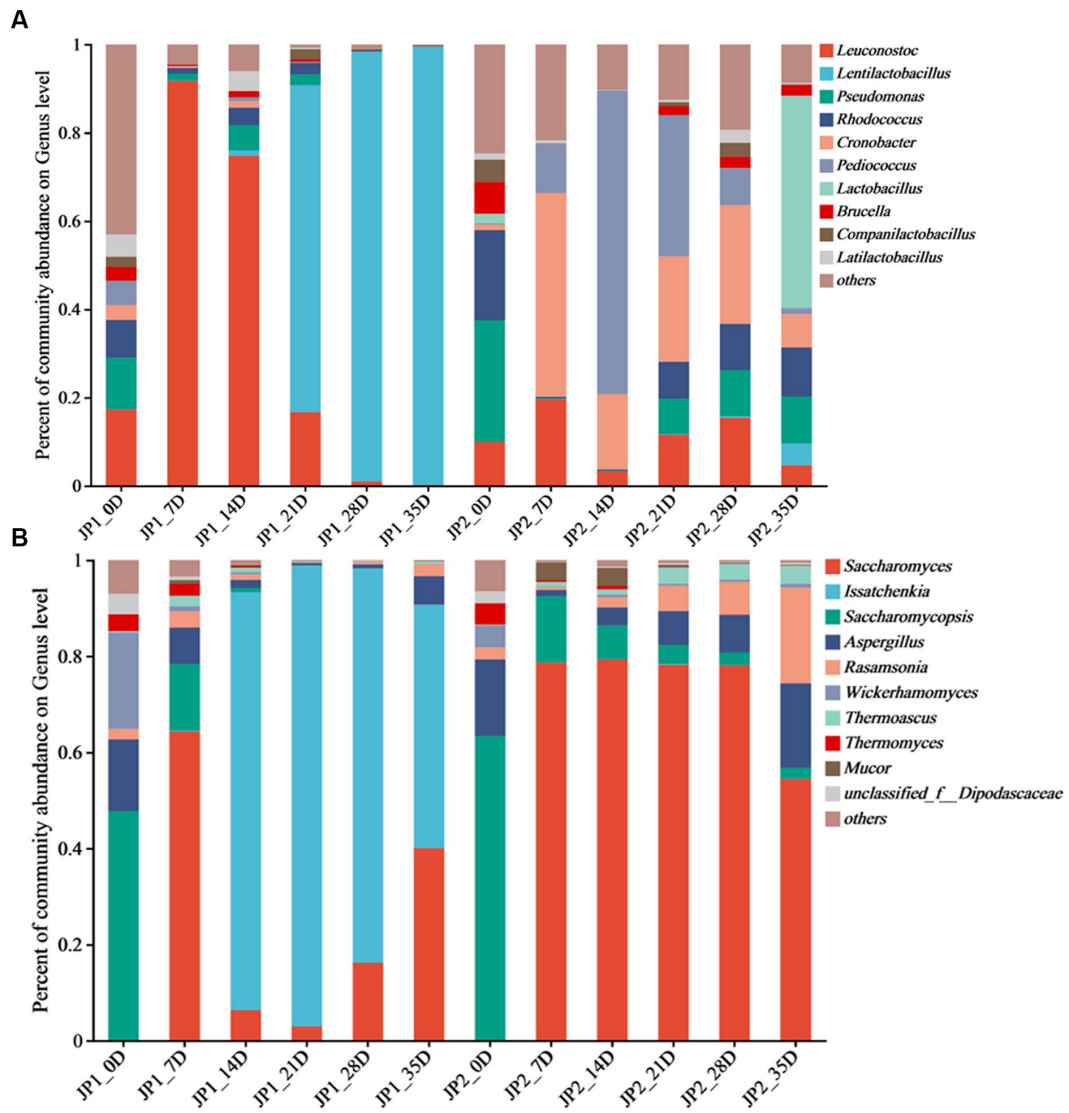
Distribution of microbial community at genus level during the fermentation of JPs. **(A)** 201 Bacteria. **(B)** Fungi.

The dynamics of the main physicochemical properties throughout the fermentation process are shown in Figure 1. During the fermentation, the water content in the two JPs modestly increased, possibly caused by the aerobic respiration of mold and yeast. Reducing sugar in JP1 presented an upward trend and reached its maximum on day 7, indicating the saccharification speed was faster than the consumption speed. After that, reducing sugar gradually decreased until the end of fermentation. It is worth noting that reducing sugar in JP1 is higher than that in JP2, due to the rich sugar content in *Hovenia acerba* (Yang et al., 2023). The initial acidity of JP1 was slightly higher than that of JP2 due to the addition of *Hovenia acerba*. In the early stage of fermentation, acidity increased rapidly, and pH presented the opposite trend due to the proliferation of acid-producing bacteria, such as *Leuconostoc* and *Pediococcus* (Figure 2). From the 7th to the 28th day, acidity and pH tend to be stable owing to the transformation of organic acids to esters through the esterification reaction, leading to the production and consumption of organic acids in dynamic equilibrium (Xu et al., 2017).

### 3.2 Volatile flavor compounds, organic acids, and polyphenols

**Figure 3:**
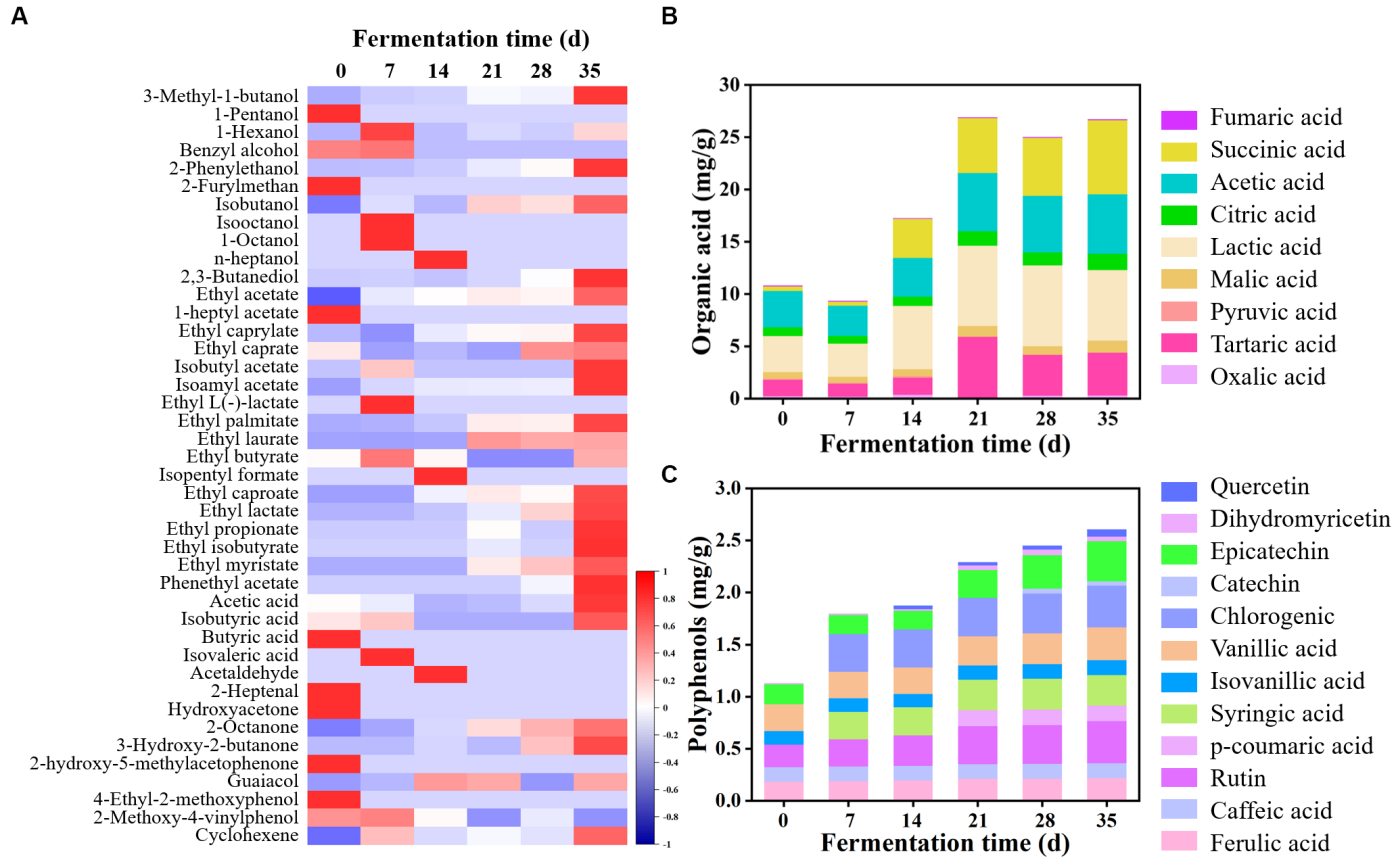
Dynamics of volatile flavor compounds, organic acids, and polyphenols during the fermentation of JP1. **(A)** Heatmap of volatile flavor compounds. The statistic of volatile flavor compounds had been produced by *z*-score. **(B)** Organic acids. **(C)** Polyphenols.

Volatile flavor compounds were detected using HS-SPME-GC–MS. As shown in Figure 3A and Supplementary Figure S1A, a total of 50 kinds of components were observed in all samples, including 14 alcohols, 20 esters, 4 acids, 2 aldehydes, 4 ketones, 3 phenols, and 3 alkenes. The number of volatile flavor compounds in JP1 was higher than that in JP2. At the beginning of fermentation, benzyl alcohol, 1-heptyl acetate, ethyl caprate, isobutyric acid, butyric acid, hydroxyacetone, 2-heptanol, and 2-methoxy-4-vinylphenol were detected only in JP1, which may be derived from *Hovenia acerba*. As the fermentation proceeded, volatile flavor compounds continuously changed. Alcohols, acids, esters, aldehydes, and ketones transformed into each other, endowing Baijiu with a unique flavor (Xu et al., 2022). Principal component analysis based on the content of volatile flavor compounds showed that the flavor structure of two JPs significantly differentiated during the fermentation (Supplementary Figure S2).

Esters were the most abundant volatile flavor compounds in two JPs, among which ethyl acetate was the dominant component. In addition, ethyl palmitate, ethyl lactate, ethyl propionate, phenyl ethyl acetate, ethyl isobutyrate, and ethyl caproate were also largely produced in JPs. It was worth noting that the content of ethyl acetate, ethyl butyrate, and ethyl caproate in JP1 was higher than that in JP2, probably because *Lentilactobacillus* and *Issatchenkia* largely existed in JP1 (Figure 2), resulting in the formation of large amounts of acids and esters (Qian et al., 2023). Higher alcohols in JP1 were higher than those in JP2, which may be derived from the hydrolysis of pectin, as well as the deamination and decarboxylation of amino acids in *Hovenia acerba* (Li et al., 2018). Research suggested that certain content of higher alcohols could increase the sense of harmony and fullness of Baijiu (Yang et al., 2014). Acetic acid was the main acid in Baijiu. As for aldehydes and ketones, 2-octanone and 3-hydroxy-2-butanone were relatively high in JP1. Furthermore, guaiacol and 4-ethyl-2-methoxyphenol also existed in both JPs.

Organic acids, one of the most important ingredients in Baijiu, not only endow Baijiu with a unique flavor but also inhibit the growth of undesired microorganisms during fermentation (Wei et al., 2020). As shown in Figure 3B and Supplementary Figure S1B, a total of nine kinds of organic acids were detected in two JPs. Organic acids were partly derived from raw materials. At the beginning of the fermentation, the total content of organic acids in JP1 and JP2 were 10.78 and 3.47 mg/g, respectively, indicating that *Hovenia acerba* is rich in organic acids. In addition, organic acids could also be produced by the metabolism of yeast and bacteria (Li et al., 2018; Sifeeldein et al., 2019). As the fermentation proceeded, organic acids increased and reached 26.68 and 24.84 mg/g in JP1 and JP2 on day 35, among which lactic acid, succinic acid, acetic acid, and citric acid were the dominant components.

Polyphenols are common bioactive substances in fermented food, exhibiting functions of anti-cancer, anti-inflammation, anti-oxidation, and anti-atherosclerosis (Stuper-Szablewska and Perkowski, 2019). As shown in Figure 3C and Supplementary Figure S1C, a total of eight kinds of polyphenols have been detected in raw materials, including ferulic acid, caffeic acid, rutin, isocyanic acid, vanillic acid, epicatechin, dihydromyricetin, and p-coumaric acid. Notably, dihydromyricetin was a unique component in *Hovenia acerba*, possessing functions such as reducing blood sugar and blood lipid, inhibiting liver cell deterioration, and preventing alcoholic liver disease (Song et al., 2022). It can also promote the growth of *Lactobacillus* and *Lentilactobacillus* (Dong et al., 2021) and inhibit the activity of *Staphylococcus aureus*, *Salmonella*, *Escherichia coli*, and *Aspergillus flavus* (Cui et al., 2021). During fermentation, polyphenols gradually increased and reached 2.60 and 2.27 mg/g at the end of fermentation, among which epicatechin, chlorogenic, vanillic acid, and rutin were the main components. Some new components were also produced, such as p-coumaric acid and catechin in JP1, and syringic acid and chlorogenic in JP2.

### 3.3 Microbial community diversity

The microbial community structure was explored by high-throughput sequencing. A total of 526 bacterial OTUs and 113 fungal OTUs were obtained according to 97% sequence homology. As the diversity analysis shown in Table 1, the coverage of all samples was above 0.9995, indicating that the sequencing depth was sufficient to fully reveal the microbial community of JPs.

**Table 1:** Microbial diversity during the fermentation.

Project	Sample	Ace	Chao	Shannon	Simpson	Coverage
Bacteria	JP1_0D	402.23	400.40	3.2974	0.0694	0.9998
	JP2_0D	274.33	272.91	2.6636	0.1387	0.9999
	JP1_7D	126.38	119.55	0.5143	0.8425	0.9997
	JP2_7D	46.95	45.25	1.9196	0.2241	0.9998
	JP1_14D	116.56	118.46	1.2267	0.5669	0.9998
	JP2_14D	57.05	48.00	1.1405	0.5014	0.9997
	JP1_21D	53.07	54.00	0.9133	0.5788	0.9996
	JP2_21D	67.98	65.00	2.1551	0.1813	0.9997
	JP1_28D	26.76	25.00	0.1626	0.9488	0.9998
	JP2_28D	86.80	82.00	2.5671	0.1155	0.9996
	JP1_35D	14.97	13.50	0.0485	0.9874	0.9999
	JP2_35D	84.90	79.55	1.9415	0.2665	0.9995
Fungi	JP1_0D	85.07	84.43	1.8555	0.2843	1.0000
	JP2_0D	64.05	68.00	1.5478	0.4253	0.9999
	JP1_7D	68.50	70.00	1.4132	0.4402	0.9998
	JP2_7D	38.21	37.60	0.7808	0.6400	0.9999
	JP1_14D	54.36	53.50	0.6274	0.7630	0.9999
	JP2_14D	74.93	66.00	0.9288	0.6368	0.9998
	JP1_21D	28.27	28.00	0.2144	0.9195	0.9999
	JP2_21D	50.13	46.91	0.9120	0.6217	0.9999
	JP1_28D	46.94	33.20	0.5575	0.7005	0.9999
	JP2_28D	52.33	51.67	0.8722	0.6236	0.9998
	JP1_35D	28.27	27.50	1.0251	0.4208	0.9999
	JP2_35D	57.11	56.67	1.2958	0.3690	0.9999

The difference in microbial community structure between two JPs during fermentation was studied using principal coordinate analysis (Supplementary Figure S3). At the beginning of the fermentation, the samples of JP1 and JP2 were close to each other, indicating that the initial microbial community structure of the two JPs was similar. As the fermentation proceeded, the microbial community structure of JP1 and JP2 began to differentiate. In JP1, the bacterial community has mainly changed from days 0 to 7 and 14 to 21. The change in fungi was observed in the early stage of fermentation. In JP2, the bacterial community structure continually changed throughout the fermentation. Fungi remained stable in the middle and late stages of fermentation.

### 3.4 Succession of microbial community structure

At the phylum level, a total of 25 bacterial phyla were observed, among which *Firmicutes*, *Proteobacteria*, and *Actinobacteriota* presented a relative abundance of more than 1% (Supplementary Figure S4A). Throughout the fermentation process, the diversity of the microbial community declined, and *Firmicutes* was the dominant phylum (Li et al., 2011).

A total of 315 bacterial genera were found at the genus level. *Leuconostoc*, *Lentilactobacillus*, *Pseudomonas*, *Rhodococcus*, *Cronobacter*, *Pediococcus*, *Lactobacillus*, *Brucella*, *Companilactobacillus*, and *Latilactobacillus* were the top 10 abundant bacterial genera (Figure 2A). In the early stage of fermentation, the bacterial community structure in the two JPs was similar. *Leuconostoc*, *Pseudomonas*, and *Rhodococcus* were the main genera. *Leuconostoc*, which commonly exists in fermented foods such as pickles, became the dominant bacterial genera with a relative abundance of 91.8% in JP1 on day 7. As fermentation proceeded, the anaerobic environment gradually formed, and the acidity increased with the accumulation of lactic acid. *Lentilactobacillus* became the dominant genus in the middle and late stages of fermentation in JP1, with a relative abundance of 99.4% on day 35. Lactic acid and a variety of antibacterial substances were produced by *Lentilactobacillus*, inhibiting the growth of pathogens and spoilage microbes and leading to a significant reduction of bacterial diversity (Cappello et al., 2017). On day 7, the main bacteria in JP2 were *Cronobacter* (46.2%), *Pediococcus* (11.2%), and *Leuconostoc* (19.6%), whose relative abundance constantly changed during the fermentation. *Lactobacillus* was the dominant genus in the late stage of fermentation. The significant difference in bacterial community structure between two JPs may be caused by fermentation substrates and conditions. The dihydromyricetin in JP1 was beneficial for the growth and metabolism of *Lentilactobacillus* and *Leuconostoc* (Dong et al., 2021).

At the phylum level, six fungal phyla were detected in the two JPs. *Ascomycota* was the dominant phylum, with a relative abundance of more than 90%, followed by *Mucoromycota* and *Basidiomycota* (Supplementary Figure S4B).

A total of 66 fungal genera were observed at the genus level, among which *Saccharomyces*, *Issatchenkia*, *Saccharomycopsis*, *Aspergillus*, *Rasamsonia*, *Mucor*, *Wickerhamomyces*, *Thermoascus*, *Thermomyces*, and *unclassified_f__Dipodascaceae* were identified as the dominant genera (Figure 2B). At the beginning of the fermentation, *Saccharomycopsis* was the predominant fungus in the two JPs, accounting for 47.7 and 63.3%, respectively, followed by *Aspergillus* and *Wickerhamomyces*. *Saccharomycopsis* can secrete amylase, protease, and β-glucosidase to promote the saccharification of starch (Chen et al., 2020). *Aspergillus*, widely distributed in cereals, with strong protein decomposition and saccharification ability, can convert starch into fermentable sugar and further translate fermentable sugar into organic acids, amino acids, and esters (Liu et al., 2021). *Wickerhamomyces*, often found in fruit skin, can improve the flavor of Baijiu (Sun et al., 2022). According to the research, these fungi are important for the production of secondary metabolites, which contribute to enhancing the flavor of the Baijiu (Huang et al., 2018). *Issatchenkia* was the dominant fungus in JP1, and *Saccharomyces* was the most important fungus in JP2 in the middle and late stages of the fermentation. Compared with *Saccharomyces*, *Issatchenkia* was more tolerant to low acidity and could degrade malic acid and citric acid. In addition, *Issatchenkia* could promote the formation of esters and aromatic compounds (Wu et al., 2012).

### 3.5 Correlation analysis of microorganisms and physiochemical properties

**Figure 4:**
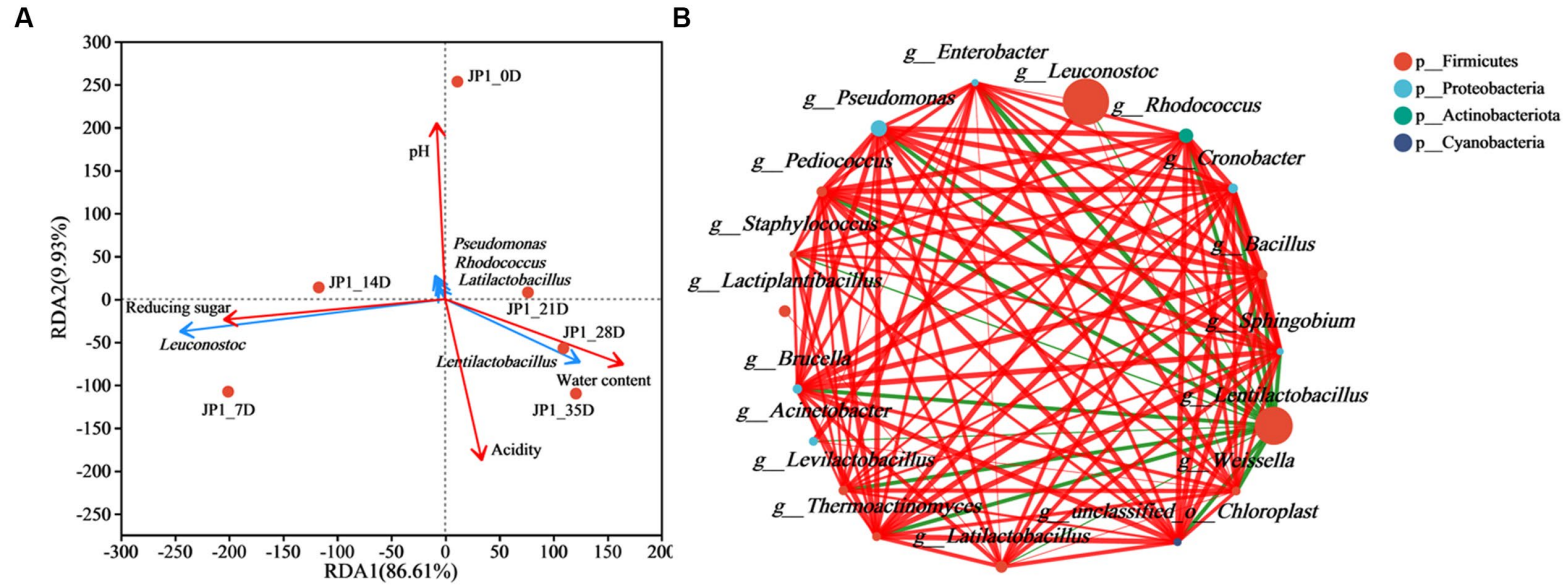
Correlation analysis of bacterial genera and physicochemical properties, and the co-occurrence network analysis of bacterial genera in JP1. **(A)** Correlation analysis of bacterial communities and physicochemical properties. **(B)** Co-occurrence network analysis of bacterial genera. Red and green represent positive and negative correlations, respectively. The thicker the line, the stronger the correlation.

Correlation analysis of microorganisms and physiochemical properties in *Hovenia acerba*-sorghum co-fermented Baijiu was conducted using redundancy analysis. As shown in Figure 4A and Supplementary Figure S5A, environmental factors exhibited distinct influences on bacteria and fungi. pH presented a great influence on microbial community distribution in the early stage of fermentation. In the middle stage of fermentation, reducing sugar became the main factor influencing the structure of the microbial community. At the end of fermentation, the microbial community structure was largely affected by water and acidity, which were consistent with the research of Xu (Xu et al., 2022). The bacterial genera *Pseudomonas*, *Rhodococcus*, and *Latilactobacillus* exhibited a positive correlation with pH (Chen et al., 2020). In addition, *Lentilactobacillus* and *Leuconostoc* presented a positive correlation between water content and reducing sugar, respectively. For fungi, *Saccharomycopsis*, *Wicherhamomyces*, and *Aspergillus* are located in the same quadrant with pH, indicating a strong correlation between them. *Saccharomyces* was related to reducing sugar due to its ability to produce various carbohydrate enzymes, especially amylase (Sun et al., 2010).

To illustrate the beneficial or antagonistic relationship among microorganisms, the Spearman correlation coefficient and *value of p* were used to analyze the interaction among microorganisms at the genus level. As the results of the bacteria shown in Figure 4B, *Lentilactobacillus*, *Enterobacter*, and *Weissella* presented a close relationship with other species. *Leuconostoc* and *Pseudomonas* exhibited a maximum positive correlation with *Acinetobacter* and *Cronobacter*, respectively. *Lentilactobacillus* showed a maximum negative correlation with *Weissella*. *Leuconostoc* can rapidly produce lactic acid and acetic acid in the early stage of fermentation, reducing the pH (Zhou et al., 2023). However, along with the fermentation, acidity and ethanol increased, and the oxygen content decreased, inhibiting the growth of *Leuconostoc*. *Lentilactobacillus* was more resistant to acidity, ethanol, and low oxygen, completing the subsequent fermentation process, which was consistent with the fact that the relative abundance change of *Leuconostoc* and *Lentilactobacillus* is described in Figure 2A (Wang et al., 2008). For fungi (Supplementary Figure S5B), *Wallemia*, *Saccharomycopsis*, *Cryptococcus_f_Tremellaceae*, *Pichia*, and *Wickerhamomyces* possessed more nodes. *Cryptococcus_f_Tremellaceae*, *Geotrichum*, and *Coniochaeta* exhibited a maximum positive correlation with *Wallemia*, *unclassified_f__Dipodascaceae*, and *Trichosporon*. *Aspergillus* presented a negative relationship with *Issatchenkia*.

### 3.6 Correlation analysis of microorganisms and organic acids, polyphenols, and volatile flavor compounds

To explore the roles of microorganisms in the formation of organic acids, polyphenols, and volatile flavor compounds during Baijiu fermentation, correlation analysis was conducted.

**Figure 5:**
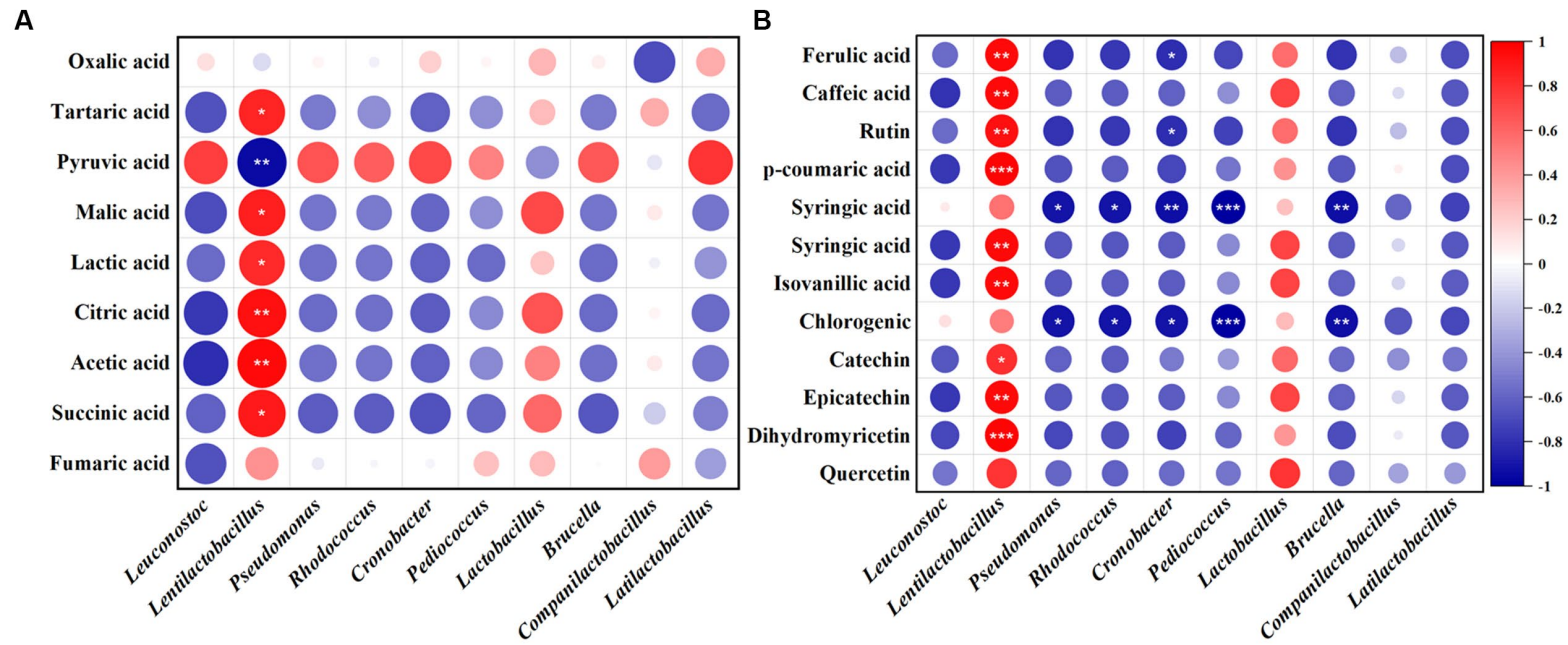
Correlation analysis of bacterial genera with organic acids and polyphenols in JP1. **(A)** Organic acids. **(B)** Polyphenols. Red and blue represent positive and negative correlations, respectively.

The relationship of bacteria and fungi with organic acids and polyphenols in JP1 is shown in Figure 5 and Supplementary Figure S6. *Lentilactobacillus* and *Issatchenkia* were positively correlated with most organic acids and polyphenols. *Lentilactobacillus* plays an important role in the formation of organic acids in fermented food, including lactic acid, acetic acid, and citric acid. *Issatchenkia* might be involved in malic–lactic acid fermentation and transform malic acid and citric acid into lactic acid with a more mellow flavor (Qiu et al., 2022). Pyruvate was positively correlated with most of the bacteria and fungi genera.

**Figure 6:**
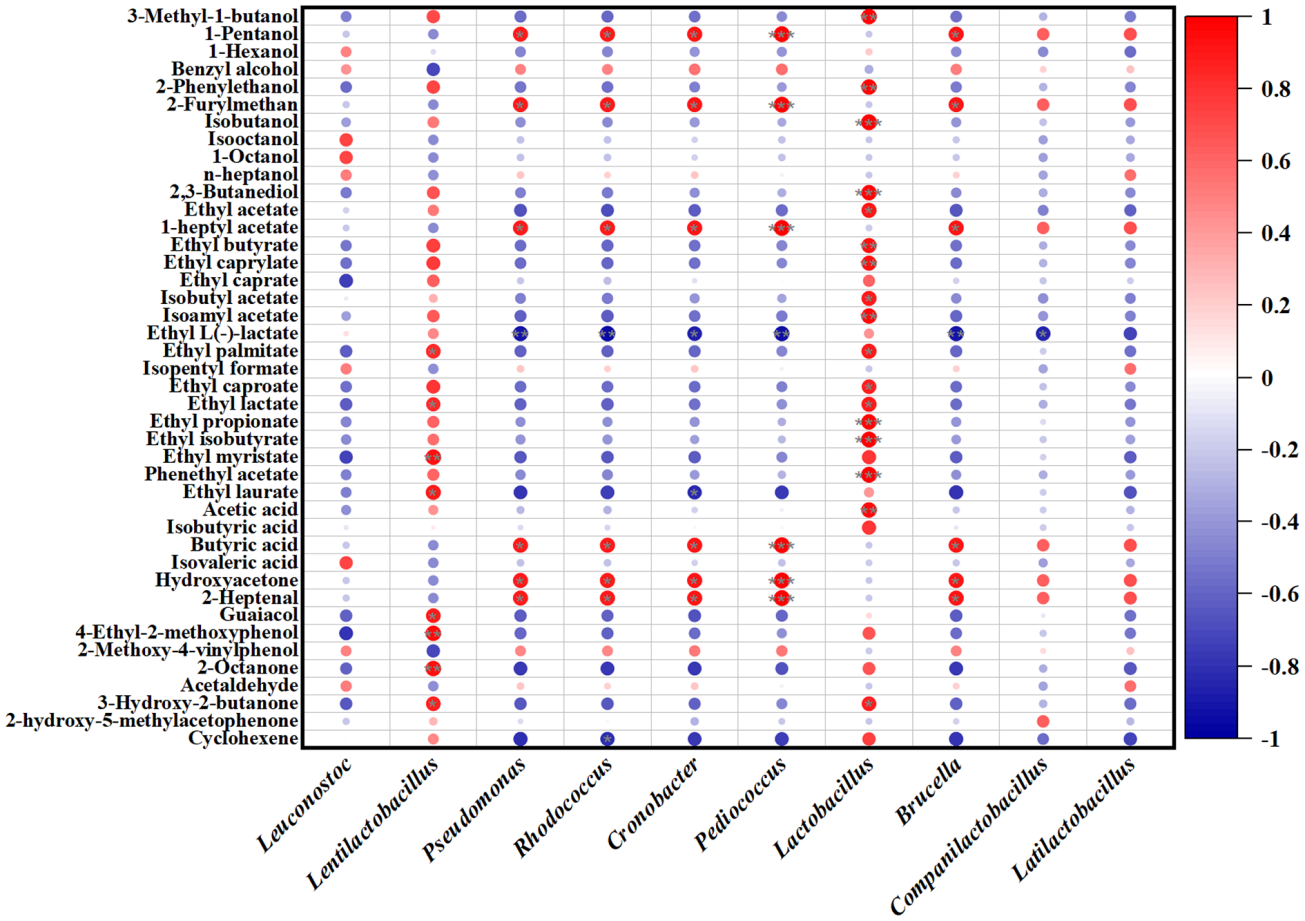
Correlation analysis of bacterial genera and volatile flavor compounds in JP1.

The correlation analysis of microorganisms and volatile flavor compounds in JP1 is shown in Figure 6 and Supplementary Figure S7. For bacteria, 1-pentanol, 2-furylmethan, 1-heptyl acetate, butyric acid, hydroxyacetone, and 2-heptanol were positively correlated with *Pseudomonas*, *Rhodococcus*, *Cronobacter*, *Pediococcus*, and *Brucella*. Ethyl myristate, 4-ethyl-2-methoxyphenol, guaiacol, and 2-octanone presented a positive correlation with *Lentilactobacillus*. *Lactobacillus* was positively related to the most volatile flavor compounds.

Fungi also play important roles in the formation of volatile flavor compounds. *Saccharomycopsis*, *Wickerhamomyces*, *Aspergillus*, *Thermomyces*, and *unclassified_f__Dipodascaccae* were positively correlated with 1-pentanol, 2-furylmethan, 1-heptyl acetate, butyric acid, hydroxyacetone, and 2-heptanol.

Compared with JP1, microorganisms presented a different influence on organic acids and polyphenols in JP2. As the relationship of microorganisms with organic acids and polyphenols shown in Supplementary Figures S8, S9, *Lentilactobacillus* and *Lactobacillus* were positively correlated with most organic acids and polyphenols. *Rasamsonia*, *Thermoascus*, and *Saccharomyces* exhibited a positive relationship with all organic acids and polyphenols, except for oxalic acid, fumaric acid, and catechin. The analysis of microorganisms and volatile flavor compounds in JP2 is shown in Supplementary Figures S10, S11. *Pseudomonas*, *Rhodococcus*, *Brucella*, *Companilactobacillus*, *Saccharomycopsis*, *Wickerhamomyces*, *Thermomyces*, and *unclassified_f__Dipodascaceae* presented positive relationships with 1-pentanol, 2-furylmethan, banana oil, 3-hydroxy-2-butanone, and 1-heptene. *Thermoascus* and *Saccharomyces* were positively related to most of the volatile flavor compounds.

From the results above, we can conclude that the different raw materials have different physicochemical properties and components. The fermentation conditions and components further affect the growth of microorganisms during the fermentation. Similarly, microorganisms can affect the production of components of organic acids, polyphenols, and volatile flavor compounds.

### 3.7 Metabolic functions of bacterial community during The fermentation

**Figure 7:**
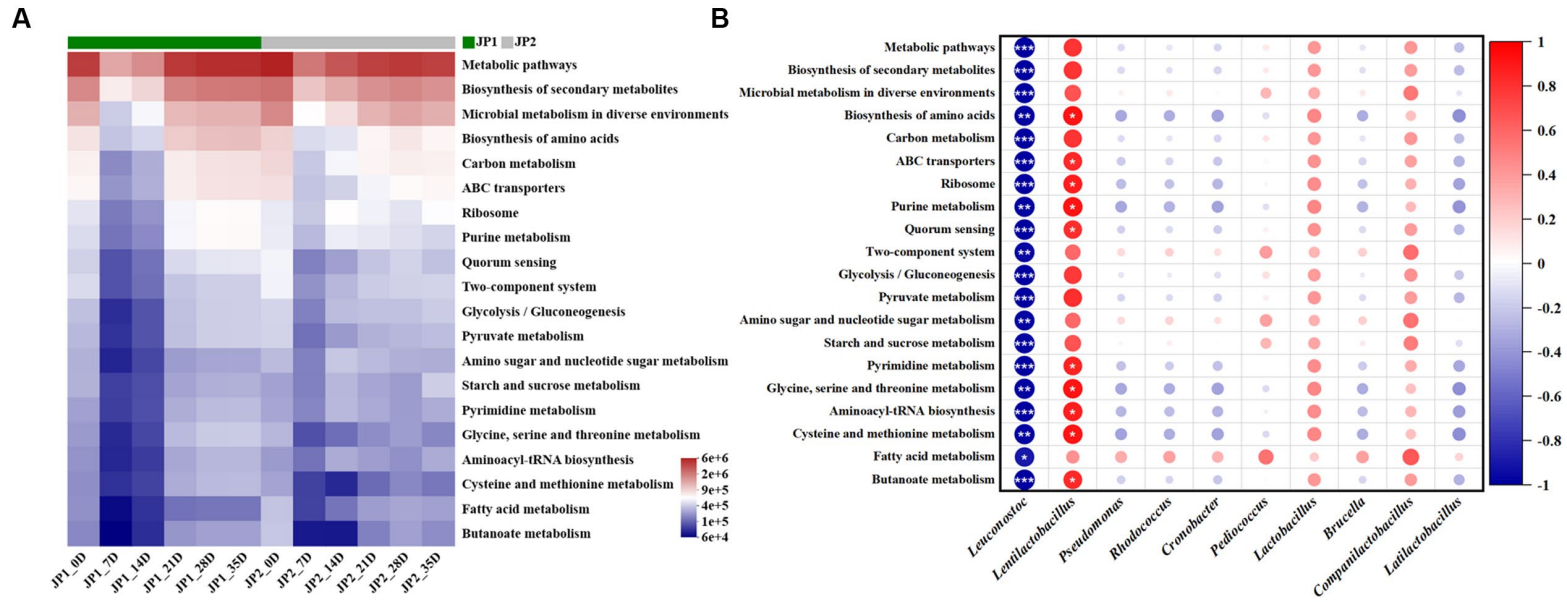
Metabolic functions prediction of the bacterial community. **(A)** Prediction of the metabolic functions of the bacterial community during the fermentation of JPs. **(B)** Correlation analysis of the main bacterial genera and metabolic pathways in JP1.

Finally, we predicted the metabolic function of the bacterial community in the two JPs with different raw materials. As shown in Figure 7, the top 20 third-order metabolic pathways in terms of abundance in the two JPs during the fermentation were screened, among which metabolic pathways, biosynthesis of secondary metabolites, microbial metabolism in diverse environments, biosynthesis of amino acids, and carbon metabolism occupied the main position. These main metabolic pathways exhibited a trend of decreasing in the early stage of fermentation and then increasing until the end of fermentation. Compared with JP2, the abundance of biosynthesis of secondary metabolites, biosynthesis of amino acids, and carbon metabolism was relatively higher than that in JP1, which may be caused by the difference in abundance and diversity of bacteria in two JPs (Figure 2). In JP1, *Lentilactobacillus* presented a positive relationship with all of the metabolic pathways as *Lentilactobacillus* was the most aboundance bacteria, which playing vital roles in the term of metabolic. For other bacteria, relatively small contribution was exhibited owing to the low abundance. In JP2, the main metabolic pathways exhibited a positive relationship with almost all bacteria except *Leuconostoc*, *Cronobacter*, and *Pediococcus* (Supplementary Figure S12). That was because the abundance and diversity of bacteria in JP2 were more balanced than in JP1.

The results above exhibited that the difference in metabolic pathways existed in the two JPs, demonstrating that the different raw materials could promote the growth of specific bacteria, which further led to the difference in metabolism.

## 4 Conclusion

In this study, microbial community diversity and its correlation with physicochemical properties, organic acids, polyphenols, and volatile flavor components of Baijiu fermented from different raw materials were comprehensively researched. In *Hovenia acerba*-sorghum co-fermented Baijiu, organic acids, polyphenols, and volatile flavor components were abundant, which improved the nutritional value and health function of Baijiu. During the fermentation, four kinds of bacterial phyla were annotated, including *Firmicutes*, *Proteobacteria*, and *Firmicutes*. *Leuconostoc* was the dominant genus at the beginning of fermentation, and *Lentilactobacillus* was the main genus in the late stage of fermentation in JP1. *Cronobacter* and *Pediococcus* were the dominant genera in JP2. *Issatchenkia* and *Saccharomyces* were the dominant fungal genera in JP1 and JP2, respectively. Correlation analysis indicated that *Lentilactobacillus* and *Issatchenkia* were core functional microorganisms that played a crucial role in the bioconversion of organic acids and polyphenols. *Pseudomonas*, *Rhodococcus*, *Cronobacter*, *Pediococcus*, *Brucella*, *Lentilactobacillus*, *Lactobacillus*, *Saccharomycopsis*, *Wickerhamomyces*, *Aspergillus*, *Thermomyces*, and *unclassified_f__Dipodascaccae* exhibited a positive correlation with the main volatile flavor components. Furthermore, the main metabolic pathways in JP1 were more active than those in JP2. Overall, the understanding of microbial diversity as well as the correlation of microorganisms with physicochemical properties, organic acids, polyphenols, and volatile flavor components can lay the foundation for sustainable, high-quality *Hovenia acerba*-sorghum co-fermented Baijiu production.

## Data availability statement

The original contributions presented in the study are included in the article/Supplementary material, further inquiries can be directed to the corresponding author.

## Author contributions

JZ: Conceptualization, Methodology, Writing – original draft. MZ: Conceptualization, Writing – original draft. JC: Methodology, Writing – original draft. YuZ: Methodology, Writing – review & editing. CX: Investigation, Writing – review & editing. QL: Data curation, Writing – review & editing. XW: Conceptualization, Writing – original draft. YD: Supervision, Writing – review & editing. YoZ: Funding acquisition, Supervision, Writing – review & editing.
