# Supplementary material for: The improvement of Hovenia acerba-sorghum co-fermentation in terms of microbial diversity, functional ingredients, and volatile flavor components during Baijiu fermentation
Source: Front Microbiol. 2024 Jan 5;14:1299917. doi: 10.3389/fmicb.2023.1299917 (PMC10797018; doi:10.3389/fmicb.2023.1299917)
Supplement: Supplementary file 1 [file Data_Sheet_1.pdf]

# supplementary material

The improvement of Hovenia acerba-sorghum co-fermentation in terms of microbial diversity, functional ingredients, and volatile flavor components during Baijiu fermentation

## Supplementary Figures

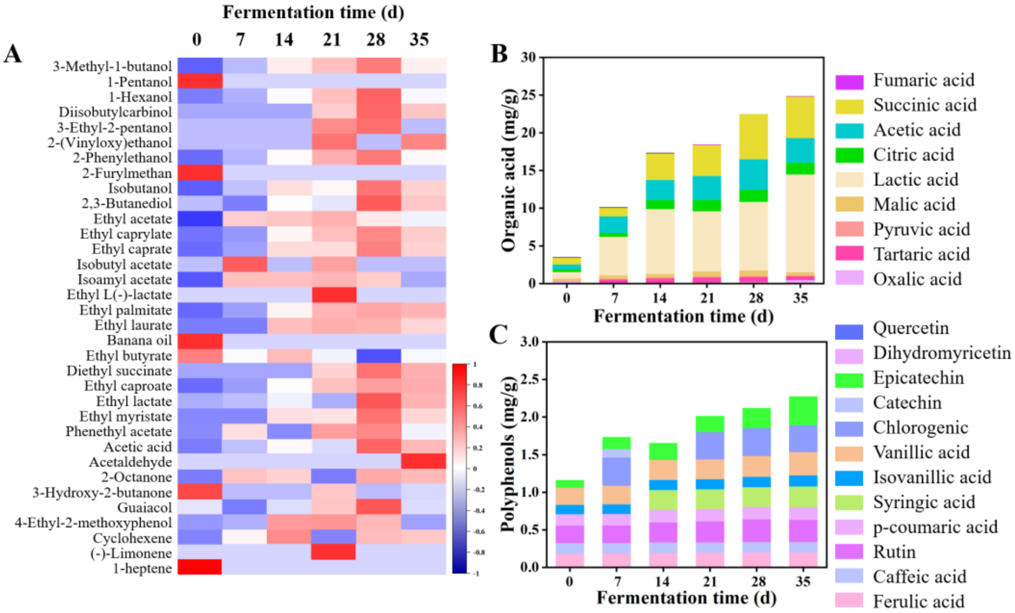


**Fig. S1**. Dynamics of volatile flavor compounds, organic acids and polyphenols during fermentation of JP2. (A) Heatmap of volatile flavor compounds. The statistic of volatile flavor compounds had been produced by z-score. (B) Organic acids. (C) Polyphenols.

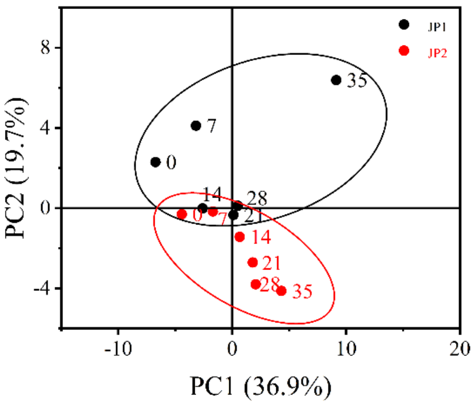


**Fig. S2**. Principal component analysis of volatile flavor compounds during fermentation of JP.

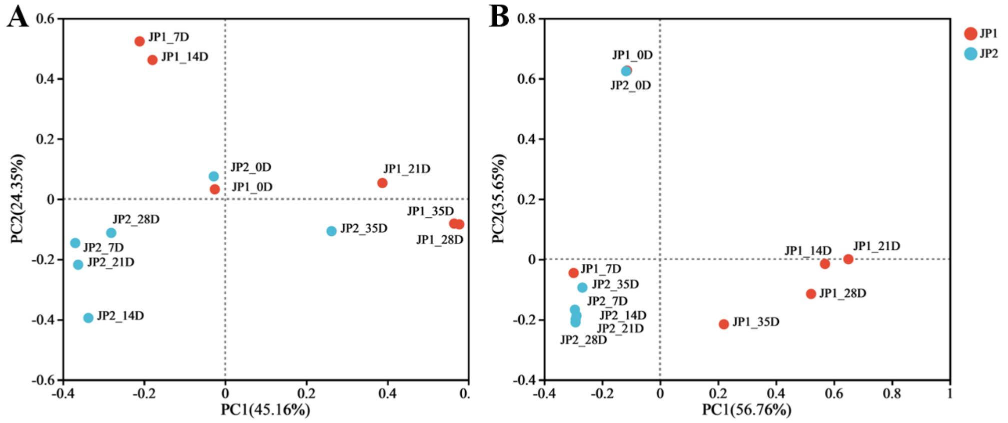


**Fig. S3**. Dynamics of the microbial community during fermentation of two JPs. Principal coordinate analysis of bacteria (A) and fungi (B).

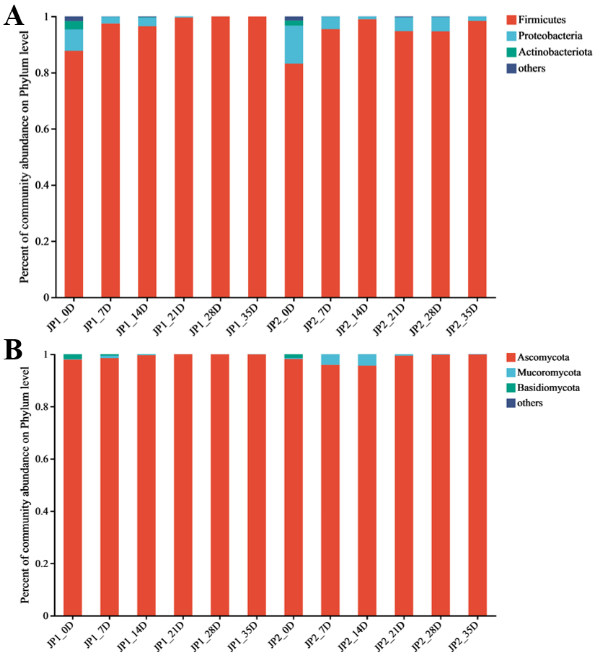


**Fig. S4**. Distribution of microbial community at phylum level during fermentation of JPs. (A) Bacteria. (B) Fungi.

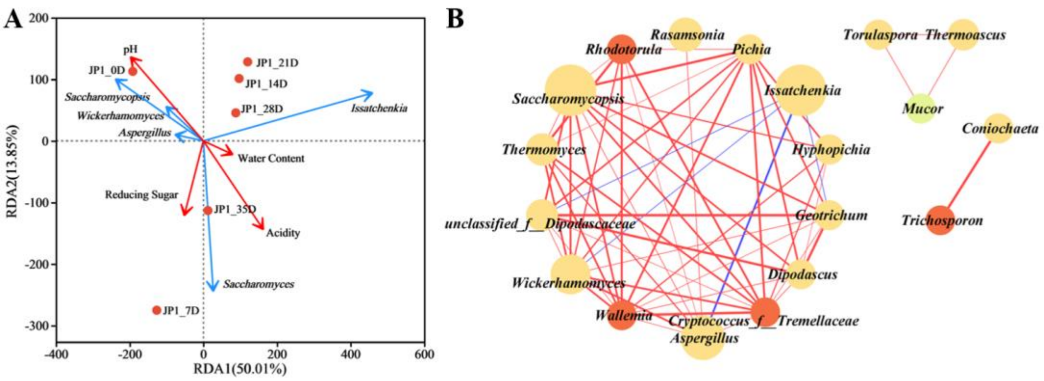


**Fig. S5**. Correlation analysis of fungal genera and physicochemical properties, and the co-occurrence network analysis of fungal genera in JP1. (A) Correlation analysis of fungal community and physicochemical properties. (B) Co-occurrence network analysis of top 20 fungal genera.

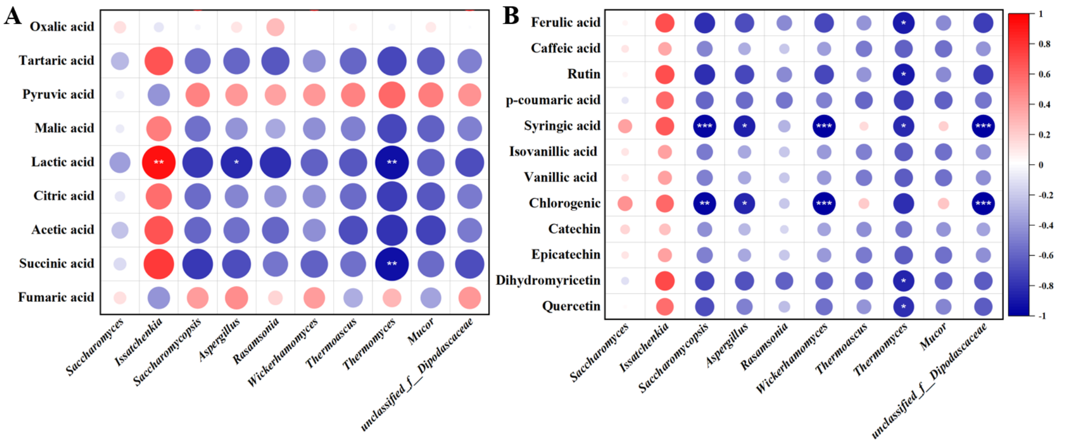


**Fig. S6**. Correlation analysis of fungal genera with organic acids and polyphenols in JP1. (A) Organic acids. (B) Polyphenols.

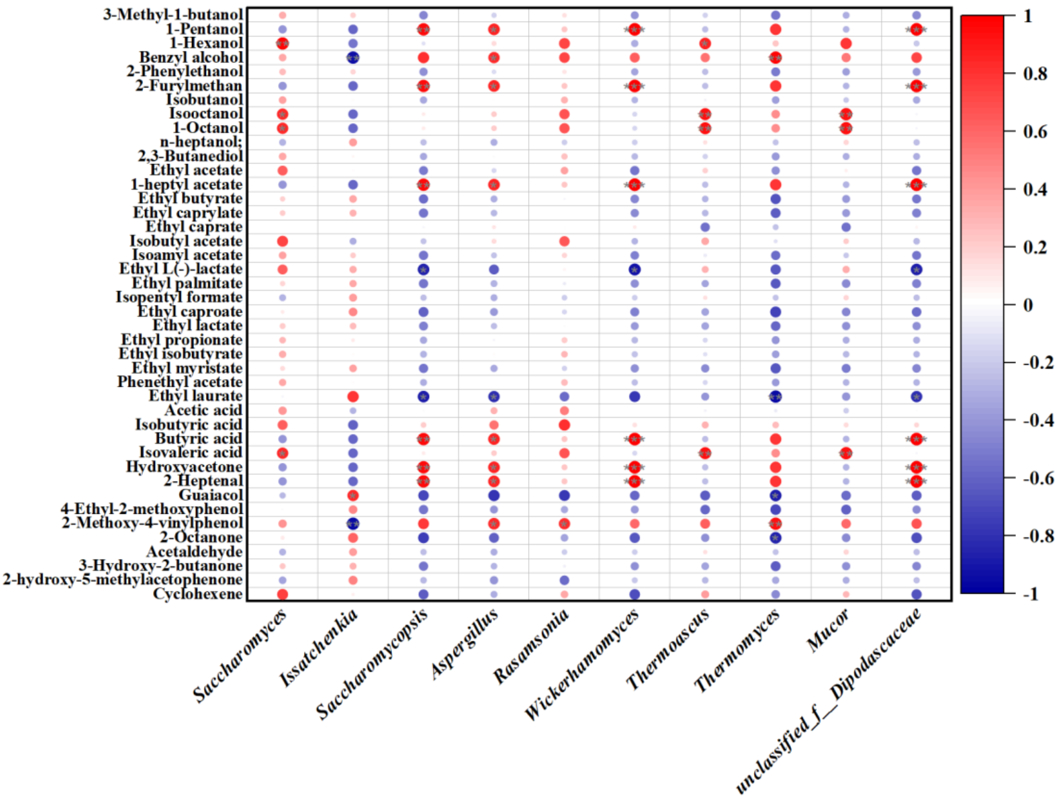


**Fig. S7**. Correlation analysis of fungal genera and volatile flavor compounds in JP1.

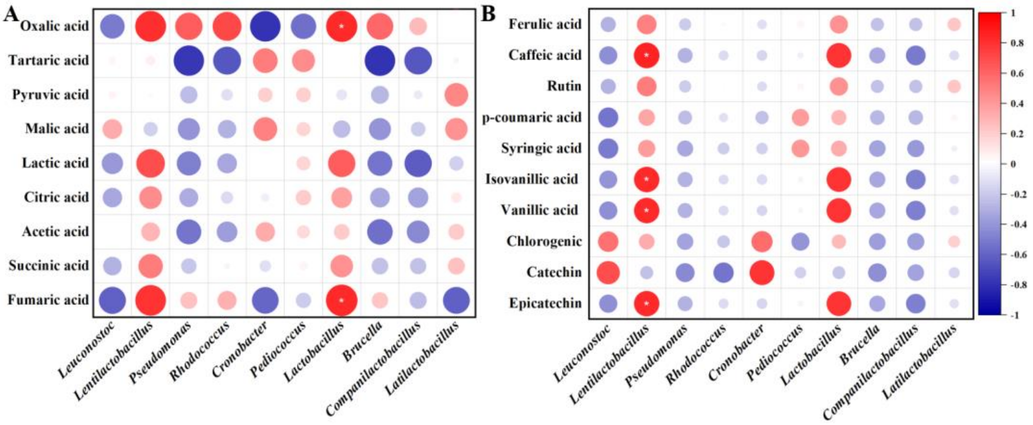


**Fig. S8**. Correlation analysis of bacterial genera with organic acids and polyphenols in JP2. (A) Organic acids. (B) Polyphenols.

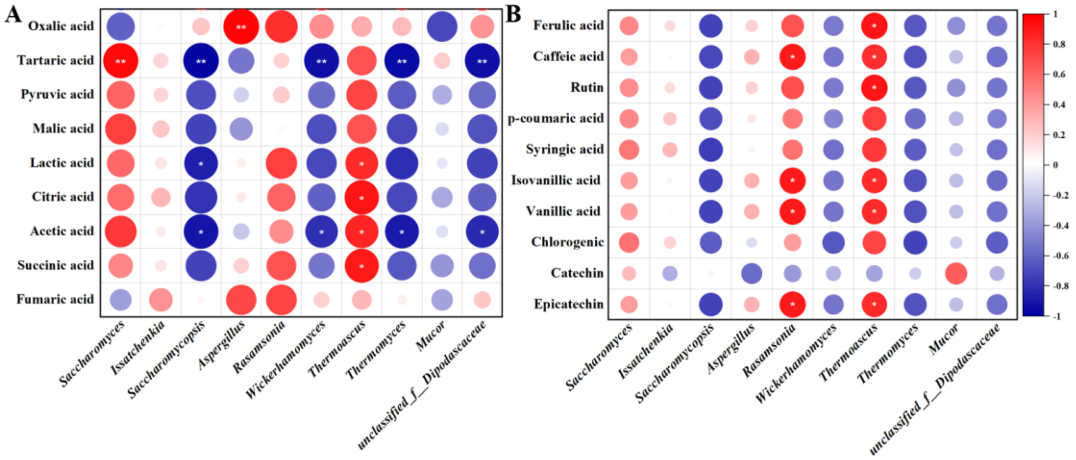


**Fig. S9**. Correlation analysis of fungal genera with organic acids and polyphenols in JP2. (A) Organic acids. (B) Polyphenols.

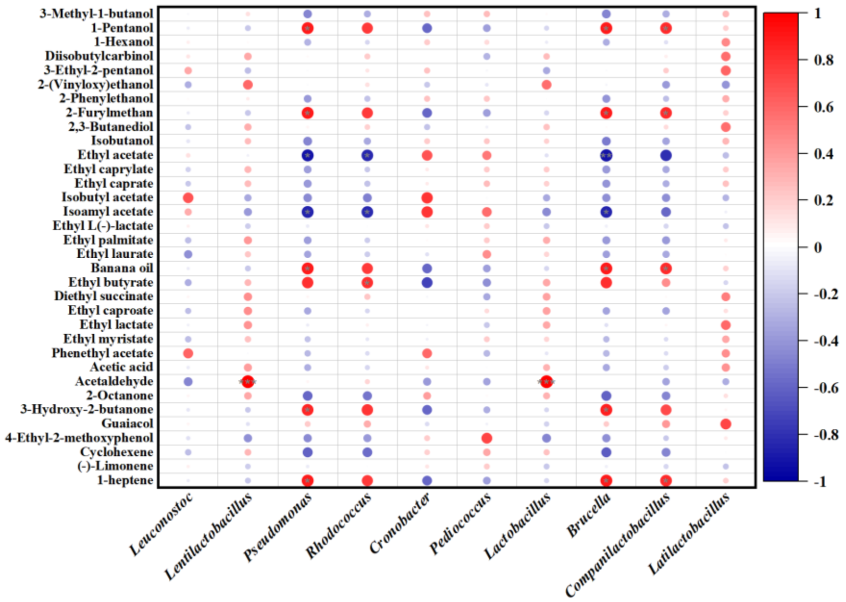


**Fig. S10**. Correlation analysis of bacterial genera and volatile flavor compounds in JP2.

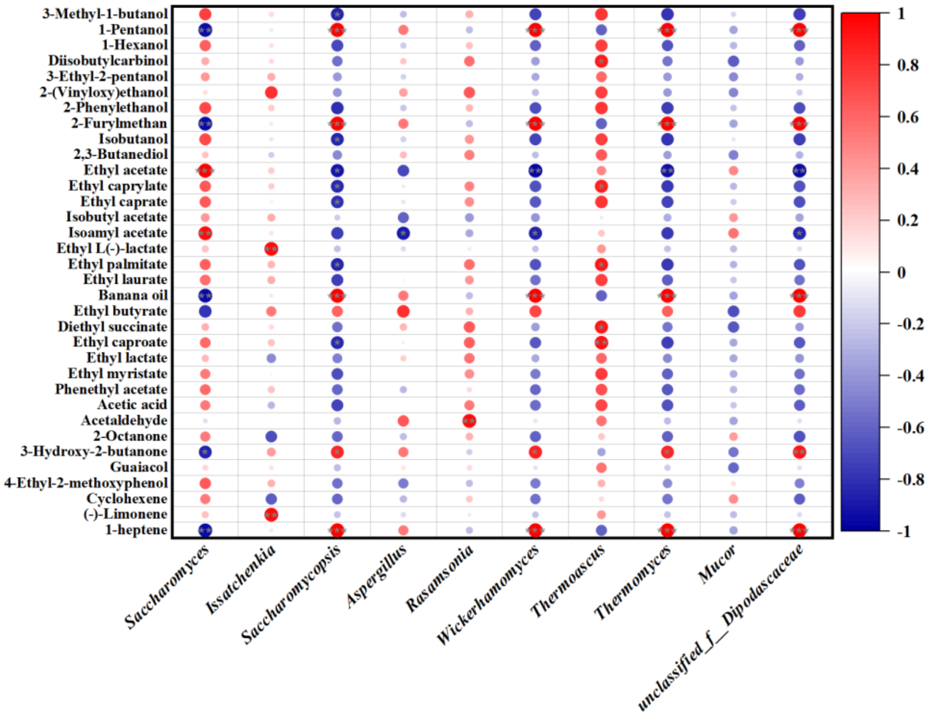


**Fig. S11**. Correlation analysis of fungal genera and volatile flavor compounds in JP2.

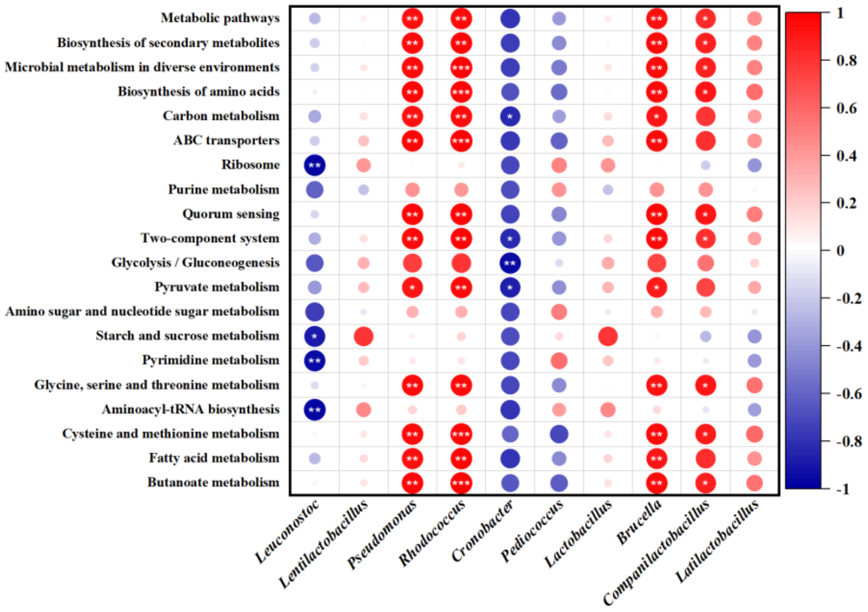


**Fig. S12**. Correlation analysis of the main bacterial genera and metabolic pathways in JP2.

# Supplementary tables

**Table S1**. Gradient elution program of high-performance liquid chromatography.

| Time（min） | Mobile phase A（%） | Mobile phase B（%） |
|---|---|---|
| 0 | 95 | 5 |
| 5-15 | 85 | 15 |
| 15-20 | 80 | 20 |
| 20-31 | 70 | 30 |
| 31-35 | 55 | 45 |
| 35-43 | 45 | 55 |
| 43-48 | 35 | 65 |
| 48-60 | 95 | 5 |
